# Internal and External Temperature Monitoring of a Li-Ion Battery with Fiber Bragg Grating Sensors

**DOI:** 10.3390/s16091394

**Published:** 2016-08-30

**Authors:** Susana Novais, Micael Nascimento, Lorenzo Grande, Maria Fátima Domingues, Paulo Antunes, Nélia Alberto, Cátia Leitão, Ricardo Oliveira, Stephan Koch, Guk Tae Kim, Stefano Passerini, João Pinto

**Affiliations:** 1 Department of Physics & I3N, University of Aveiro, Campus de Santiago, 3810-193 Aveiro, Portugal; micaelnascimento@ua.pt; 2 Helmholtz Institute Ulm (HIU) Electrochemistry I, Helmholtzstraße 11, 89081 Ulm, Germany and Karlsruhe Institute of Technology (KIT), P.O. Box 3640, 76021 Karsruhe, Germany; lorenzogrande.87@gmail.com (L.G.); stephan.koch@kit.edu (S.K.); guk-tae.kim@kit.edu (G.T.K.); stefano.passerini@kit.edu (S.P.); 3 Instituto de Telecomunicações and I3N, University of Aveiro, Campus de Santiago, 3810-193 Aveiro, Portugal; fatima.domingues@ua.pt; 4 Instituto de Telecomunicações and Department of Physics & I3N, University of Aveiro, Campus de Santiago, 3810-193 Aveiro, Portugal; pantunes@ua.pt (P.A.); catia.leitao@ua.pt (C.L.); jlp@ua.pt (J.P.); 5 Instituto de Telecomunicações and Centre for Mechanical Technology and Automation, Department of Mechanical Engineering, University of Aveiro, 3810-193 Aveiro, Portugal; nelia@ua.pt; 6 Instituto de Telecomunicações, Campus de Santiago, 3810-193 Aveiro, Portugal; oliveiraricas@av.it.pt

**Keywords:** Embedded sensors, Li-ion batteries, temperature monitoring, performance, safety

## Abstract

The integration of fiber Bragg grating (FBG) sensors in lithium-ion cells for in-situ and in-operando temperature monitoring is presented herein. The measuring of internal and external temperature variations was performed through four FBG sensors during galvanostatic cycling at C-rates ranging from 1C to 8C. The FBG sensors were placed both outside and inside the cell, located in the center of the electrochemically active area and at the tab-electrode connection. The internal sensors recorded temperature variations of 4.0 ± 0.1 °C at 5C and 4.7 ± 0.1 °C at 8C at the center of the active area, and 3.9 ± 0.1 °C at 5C and 4.0 ± 0.1 °C at 8C at the tab-electrode connection, respectively. This study is intended to contribute to detection of a temperature gradient in real time inside a cell, which can determine possible damage in the battery performance when it operates under normal and abnormal operating conditions, as well as to demonstrate the technical feasibility of the integration of in-operando microsensors inside Li-ion cells.

## 1. Introduction

Lithium-ion batteries have a widespread use as power sources in portable electronics, as well as in hybrid and pure electric vehicles, due to their high specific energy, long life cycle and low self-discharge [1,2]. The first and utmost challenge in designing a Li-ion battery system is to ensure its inherent safety under both normal and abuse operating conditions. To this goal, knowledge of the internal thermal behavior is therefore critical [3,4,5]. The main underlying concern is related to the significant temperature variation under high charge/discharge rates [2]. The excess heat generated during over-charge/discharge or in the presence of short circuits may cause irreversible damage in cells and eventually lead to explosion or combustion [6,7].

It is known from the literature that the temperature near the positive electrode is higher than that near the negative one, due to the lower electrical conductivity of most cathode active materials [4]. In order to develop a satisfactory thermal management strategy and to increase the performance and lifetime of Li-ion batteries, it is essential to monitor and manage both the internal and external temperature [8,9]. Normally, the thermal monitoring of batteries is performed on their surface through the use of thermocouples or electro-mechanical sensors [10,11].

Internal monitoring, however, is challenging due to the chemically aggressive and electrically noisy environment, for which sensors with low invasiveness, mechanical robustness, immunity to electromagnetic radiation, and resistance to corrosion are required. Sensors based on fiber Bragg gratings (FBG) are an effective method to perform both static and dynamic measurements of temperature, pressure, refractive index, strain, and bending [12]. FBG-based sensors have the advantages of being very small, flexible, immune to electromagnetic interference and electrostatic discharge and also present multiplexing capabilities. All these features make them a suitable solution for the monitoring of lithium batteries and fuel cells, as previously explored in [5,13,14,15,16]. FBG sensors have also been used to monitor the strain evolution of electrodes in lithium-ion batteries [17].

In this study, the in-operando monitoring of a Li-ion cell’s internal and external temperature variations, using FBG sensors, is presented. The analysis of the internal temperature evolution during galvanostatic cycling at different C-rates provides a step forward in the understanding and safety improvement of future Li-ion battery systems.

## 2. Experimental Setup and Testing

### 2.1. Silica Fiber Stability Test

To test the silica fibers chemical stability immersed in conventional Li-ion battery electrolytes, some 2–3 cm long fiber samples were stored in PE vials together with a solution of LiPF_6_ in 50:50 wt. % EC: DMC (LP30, Sigma Aldrich, Munich, Germany). The LiPF_6_ salt contained in the electrolyte is known to react with water impurities and form hydrofluoric acid (HF), which has both a detrimental effect on the battery performance and poses a hazard risk. Given the reactivity of HF towards silicon oxide, which the FBG sensors are constituted of, small amounts of water were added to probe the sensors’ chemical reactivity and their degree of dissolution into the cell environment. Deionized water (Milli-Q, Merck Millipore, Billerica, MA, USA) was added in ppm (100, 500 and 1000) amounts to simulate different levels of electrolyte contamination. The amount of dissolved Si was determined by means of ICP-OES (5100, Agilent Technologies, Santa Clara, CA, USA).

### 2.2. Fiber Bragg Grating Sensors

The FBG sensors were inscribed on commercially available photosensitive optical fiber (Fibercore PS1250/1500) by the phase mask method. The UV radiation system used was a pulsed excimer laser (KrF) (Industrial BraggStar, Coherent), emitting at a wavelength of 248 nm, 4 mJ/pulse (20 ns duration) and 500 Hz repetition rate. Two different fiber cables were prepared, each with two 0.3 cm length FBGs, spaced by 2 cm. The Bragg wavelength variations were monitored using an interrogation system (sm125-500, Micron Optics Inc., Atlanta, GA, USA) with a sample rate of 2 Hz and a wavelength accuracy of 1 pm.

### 2.3. Li-Ion Cell Assembly and Microsensor Integration

Li-ion pouch cells were assembled using a commercial lithium iron phosphate (LFP, Clariant, Muttenz, Switzerland) cathode (91:4:5 LFP:Super C65:JSR, 12 mg·cm^−2^ active mass loading) and a graphite anode (92:3:5 SBG-1:Super C65:CMC, supplied by SGL Carbon, 5.6 mg·cm^−2^ active mass loading) with an active area of 16 cm^2^, following a procedure already described in literature [18]. Aluminum and nickel tabs were used as cathode and anode current collectors, respectively. The Li-ion pouch cell assembly was carried out inside the dry room (relative humidity < 0.1 % at 20 °C), where all materials were stored prior to usage. Two sheets of a single layer polyolefin membrane (Hipore SV718, 10 µm, Asahi Kasei, Tokyo, Japan) drenched in a 1 M solution of LP30 were used as separator. The cells showed a capacity at 1C of 20 ± 1 mAh.

The internal FBG sensors were placed between the two separators layers, at the center of the electrochemically active area and near the tab-electrode connection, and named IC (Internal Center) and IT (Internal Tab-electrode), respectively. The Li-ion pouch cells were heat-sealed under vacuum.

**Figure 1:**
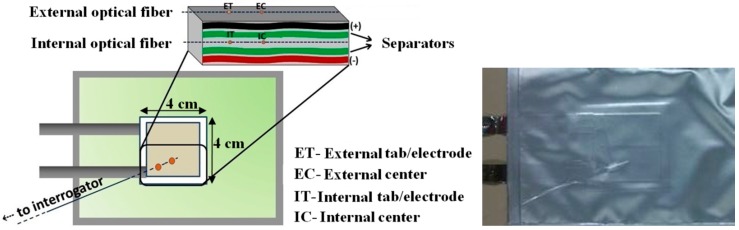
Schematic diagram of internal and external FBG sensors positions. Photograph of a pouch cell with the embedded sensors.

The external sensors were laid down in direct contact with the surface of the pouch cell, parallel to the above mentioned internal ones and named EC (External Center) and ET (External Tab-electrode), as presented in Figure 1. To increase both the contact area and the thermal conductivity, a thermal paste (Amasan Heat transfer compounds, T12) was used to attach them to the pouch cells. It should be noted that, as the cells’ thickness is very small (~1 mm), any possible strain variations were considered null, with temperature variations being the only source of the Bragg wavelength oscillations. The tests were repeated on two different cells and similar results were obtained.

### 2.4. Thermal Calibration of FBG Sensors

The FBG sensors were calibrated on a thermal chamber (Model 340, Challenge Angelantoni Industry) between 10 °C and 35 °C, in 5 °C steps. Sensitivities of 8.55 ± 0.12 pm/°C (*R*^2^ = 0.999) and 8.25 ± 0.12 pm/°C (*R*^2^ = 0.994), for ET and EC, respectively, were obtained for the external FBG sensors. To the internal FBG sensors, sensitivities of 10.24 ± 0.10 pm/°C (*R*^2^ = 0.992) and 10.27 ± 0.10 pm/°C (*R*^2^ = 0.983) were determined for IT and IC, respectively.

### 2.5. Electrochemical Testing

**Figure 2:**
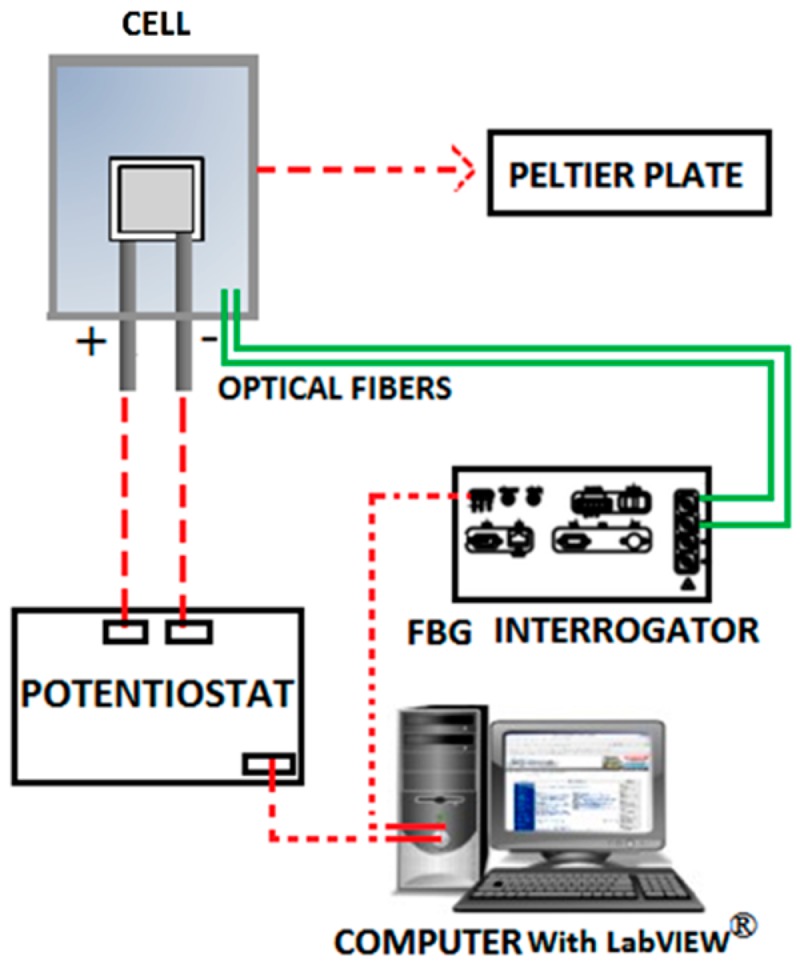
Experimental setup diagram.

The cycling tests were performed using a potentiostat/galvanostat (SP-150, Bio-Logic) at different C rates (1C, 2C, 5C and 8C). The influence of the room temperature variations was reduced using a Peltier plate connected to a temperature controller (5305 TEC Source), thus maintaining the battery at the selected temperature 20.0 ± 0.5 °C. The corresponding experimental setup is illustrated in Figure 2.

The subsequent step was the identification of the FBG sensors response to different electrochemical inputs. For this reason, the assembled cells were subjected to a cycling protocol involving a series of differing galvanostatic/potentiostatic and open circuit voltage steps, presented in Table 1.

## 3. Results and Discussion

### 3.1. Silica Fiber Chemical Inertness Study

Four polyethylene (PE) vials containing differing amounts of deionized water (0, 100, 500 and 1000 ppm) were analyzed. By the amount of Si dissolved from the fiber into the LP30 electrolyte after two weeks of storage, only small Si amounts were detected through ICP-OES (0.5–1.1 wt. %). Assuming uniform fiber dissolution, this only corresponds to the removal of a few atomic layers from the fiber surface. The optical fibers were also inspected under the microscope and no changes were observed. Taking this into consideration, the FBG sensor sensitivity and response is not expected to be altered, since the Bragg grating itself is recorded at the fiber core. A possible small attack of the cladding would not influence the FBG signal; hence, the findings confirm the suitability of glass fiber–based sensors to be used in a Li-ion pouch cell environment.

### 3.2. Analysis of the FBG Sensors Response under Different Operating Conditions

**Figure 3:**
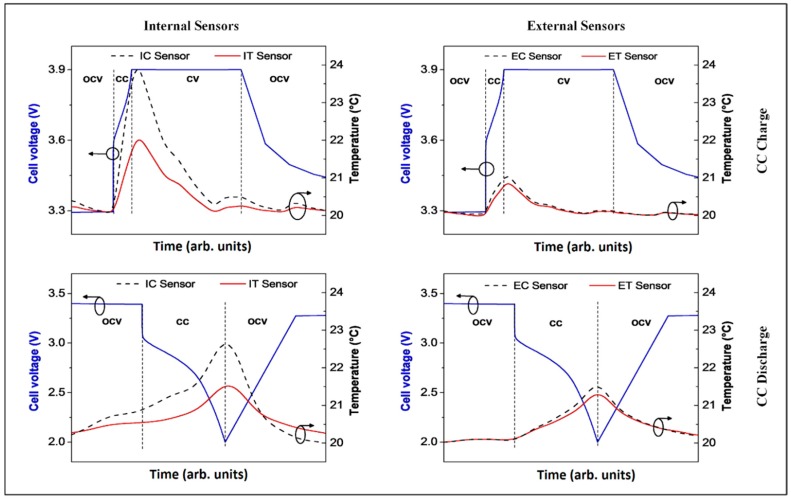
Temperature changes observed with all four sensors before, during and after a CC discharge or CCCV charge half cycle (C-rate was 5C) followed by an OCV step.

The typical response of FBG sensors during constant current (CC) discharge and constant current constant voltage (CCCV) charge half-cycles is shown in Figure 3. It is clear that the temperature rises steadily during CC charge as well as CC discharge. It is also worth noting the presence of at least two shoulders in the peak related to the CC discharge, possibly related to the different staging levels of graphite during intercalation. As soon as the applied current is lowered during the subsequent open circuit voltage (OCV) and constant voltage (CV) charge step, the temperature returns to the initial value. The baseline of the sensor signal was experimentally found to vary by ± 0.1 °C in all experiments. The origin of those slight fluctuations is not very clear, but is assumed to be correlated with external temperature variations (imperfect temperature conditioning due to the Peltier element only) and strain signals, which are neglected in this study. The temperature increase during CC charge and discharge was observed to be similar to CC discharge.

However, there is a strong difference of the ΔT values detected by internal vs. external sensors (Figure 3, left-hand side vs. right-hand side). The internal sensors directly measure the heat generated inside the battery. The external sensor’s signal was not only found to be slightly delayed with respect to the internal signal, but also the observed temperature variations are significantly lower outside the pouch bag due to the heat dissipation to the outside. Still, the general trend observed with the internal sensors is also detected by the external ones (heating during CC vs. relaxation during CV steps and at OCV).

The sensors implemented in the cell recorded a larger ΔT (4.0 ± 0.1 °C), while only 1.5 ± 0.1 °C was observed for external measurements at the center of the active area (IC vs. EC). This proves that, even in the thin single-layer cells studied here, heat dissipation does not happen immediately and, hence, is not negligible [19]. Thus, internal temperature measurements provide much more insight and a more reliable basis for the modeling of the thermal behavior of Li-ion cells. Cells used commercially, especially for mobile device applications, are rarely under the OCV condition, but are often subjected to continuous cycling. In fact, strong heating of the batteries inside smartphones is an everyday experience. This is not only unpleasant, but also deleterious to the cell life as high temperatures favor electrolyte decomposition. A better understanding of the temperature variations in Li-ion cells under heavy-duty cycling, accessible with the sensors presented in this work, is key for the improvement of cell components as well as battery management systems.

**Figure 4:**
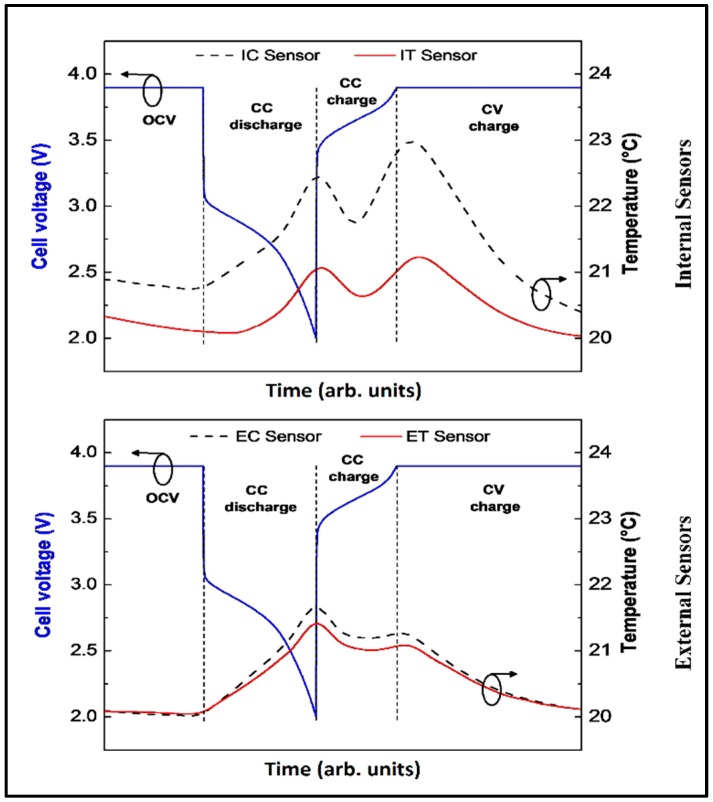
Temperature changes observed with the four sensors during cycling with a CC discharge followed directly by a CCCV charge (C-rate was 5C).

Figure 4 shows the temperature changes upon CC discharge followed directly by CCCV charge. The temperature curve of the initial CC discharge is the same as that shown before in Figure 3. However, a direct CC charging does not lead to an ongoing temperature rise. In contrast, reversing the current induces an initial temperature drop followed by another increase after a short time, resulting in an overall higher ΔT (3.0 ± 0.1 °C) than that observed during OCV separated half-cycles. During the CV charge step, where the current density decays exponentially, the temperature falls back to the surrounding temperature (controlled with the Peltier element). It can safely be assumed that this peculiar shape of the temperature curve is correlated with the concentration gradient inside the Li-ion cell. A relaxation of the gradient due to current reversing (from discharge to charge) or less current density (CV step) undoubtedly leads to thermal relaxation, i.e., faster heat dissipation than generation.

**Figure 5:**
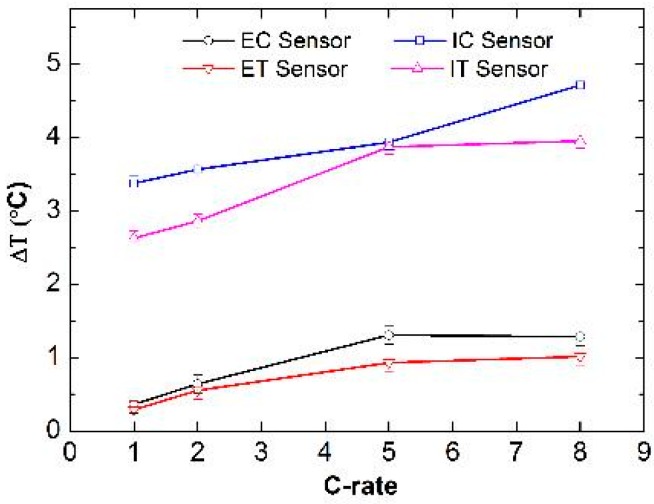
Correlation of the C-rate with the maximum ΔT recorded during cycling.

To further investigate the role of the current density, which determines the extent of cell polarization, a C-rate test was performed, comprising a series of five cycles (CC discharge + CCCV charge) at each C-rate. Figure 5 shows the correlation of the C-rate with the maximum ΔT recorded during cycling.

As Figure 5 highlights, the proximity of the internal sensors to the areas where electrochemical processes occur and heat is generated yields ΔT values that are higher than those recorded by the external sensors. The latter are unable to detect variations as high as 4.0 °C in this test setup.

The cells studied in this work were sealed inside an aluminum pouch bag foil much larger than the cell active area (100 cm^2^ vs. 16 cm^2^). It is possible that the heat generated inside the cell, which is directly recorded by the internal sensors, is dissipated over the whole pouch bag, leading to the smaller temperature changes observed with the external sensors. However, the difference in absolute ΔT values for internal and external measurements is expected to be higher for commercial cells with a high degree of electrode layer stacking, hence a more difficult way for heat dissipation. Optimized cell packaging (both casing materials and cell arrangement in full battery packs) and active cooling (especially for large batteries such as those in automotive applications) are therefore crucial to avoid dangerous temperatures inside Li-ion battery cells. In addition, permanent and immediate control of the internal temperature, as is feasible with the FBG sensor technology presented in this work, offers the possibility of the safe operation of Li-ion batteries by early detection of heat generation. Such direct temperature control is not possible with any other existing technology.

**Figure 6:**
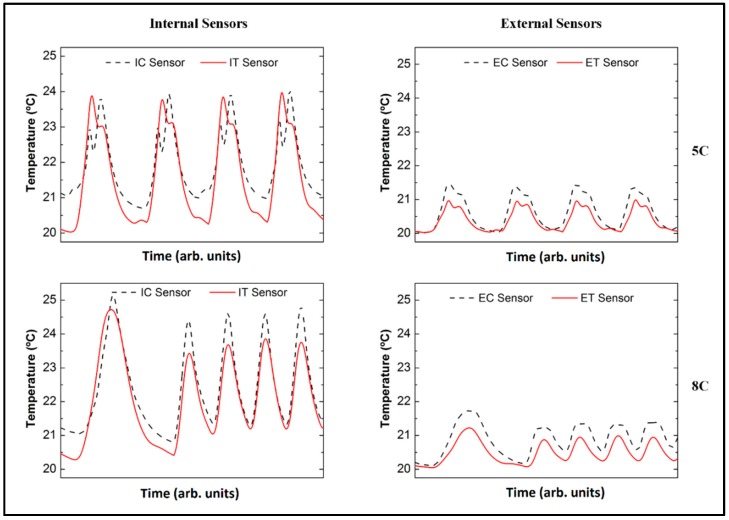
Temperature variations observed internally and externally at 5C and 8C.

In Figure 6, the temperature variations observed internally and externally at 5C and 8C, respectively, are illustrated. By comparing the FBG signals, no time delay related to the temperature variations is observed for any of the sensors, indicating that the response is always nearly instantaneous, irrespective of the positioning. Additionally, while it is still possible to distinguish two ΔT peaks corresponding to discharge and charge at 5C, respectively (Figure 6, top), at 8C these two features are merged into one single broad peak (Figure 6, bottom). This might be a direct result of the short time that separates the establishment of the two opposite concentration gradients. However, it is possible that the short cycle duration at 8C (caused by the poor rate capability of the tested cells) does not actually lead to a full establishment of the concentration gradient, hence mitigating possible relaxation effects upon the current reversing.

During the first cycle at 8C, the maximum temperature variation of 4.0 ± 0.1 °C was detected by the IC sensor, while the IT sensor detected a value of about 4.7 ± 0.1 °C. Externally, the values detected by ET and EC are in the range of 1.5 ± 0.1 °C, indicating that the aluminum pouch bag foil allows the cell to equilibrate with the ambient temperature.

## 4. Conclusions

FBG sensors were successfully integrated in Li-ion pouch cells in order to monitor temperature changes during electrochemical testing at different C-rates (1C, 2C, 5C and 8C). Two different areas (the tab-electrode connection and the center of the electrochemical active area) were monitored, both on the inside and the outside of the pouch cell. The changes in temperature showed a direct correlation with the applied current gradient, with the highest peaks being detected always at the end of charge and discharge. This is in accordance with the fact that, over both charge and discharge, Li ions migrate inside the cell to establish a concentration gradient, which generates heat as a function of the applied current. The results show that, internally, the cell temperature increased as much as 4.7 ± 0.1 °C. This outcome needs to be taken into account for battery modeling and battery management system purposes, since cell damage caused by overheating is an important reason for capacity fading in Li-ion batteries and thermal runaways are the major source of safety concerns. The FBG sensors were able to detect such temperature changes with a superior response rate, making them useful tools for failure detection in batteries. Their low invasiveness and high tolerance to the chemically aggressive environment inside Li-ion batteries makes them an interesting possibility for integration in commercial Li-ion cells as well as for research purposes for the in-situ study of temperature variations. The goal of the study was to show the functionality of the sensor and the positioning for monitoring the internal and external temperature variations in the active area of lithium batteries. In the near future the authors intend to perform an extension of this work on packs of lithium cells. A relationship between internal and external temperatures either through tables or equations is also expected to be established.

## Figures and Tables

**Table 1:** Electrochemical test protocol.

(1)	Constant Current Constant Voltage (CCCV) charge
(2)	Two cycles each composed of Constant Current (CC) discharge followed by CCCV charge
(3)	Open Circuit Voltage (OCV)
(4)	CC discharge
